# Omental Infarction Mimicking Cholecystitis

**DOI:** 10.1155/2015/687584

**Published:** 2015-02-09

**Authors:** David Smolilo, Benjamin C. Lewis, Marina Yeow, David I. Watson

**Affiliations:** ^1^Department of Surgery, Flinders Medical Centre, Bedford Park, SA 5042, Australia; ^2^Department of Clinical Pharmacology, Flinders University School of Medicine, Bedford Park, SA 5042, Australia

## Abstract

Omental infarction can be difficult to diagnose preoperatively as imaging may be inconclusive and patients often present in a way that suggests a more common surgical pathology such as appendicitis. Here, a 40-year-old Caucasian man presented to casualty with shortness of breath and progressive right upper abdominal pain, accompanied with right shoulder and neck pain. Exploratory laparoscopy was eventually utilised to diagnose an atypical form of omental infarction that mimics cholecystitis. The vascular supply along the long axis of the segment was occluded initiating necrosis. In this case, the necrotic segment was adherent with the abdominal wall, a pathology not commonly reported in cases of omental infarction.

## 1. Introduction

Omental infarction is a rare cause of acute abdominal pain, with a reported incidence of approximately 0.3%, and is found at 0.1% of laparotomies for acute abdominal pain [1]. It is most common in the 40–50-year-old age group, the frequency in men is twice that of women, and only 15% of reported cases occur in the paediatric population [1, 2]. Omental infarction may be primary (idiopathic) if there is no identifiable cause, or secondary to other pathologies. It may arise following omental torsion, which can be precipitated by postoperative adhesions, tumour, hernia, cyst, localised inflammation, or trauma. Causative factors not associated with torsion include vasculitis, hypercoagulability, and polycythaemia [3]. The right-sided omentum is more commonly involved (up to 90% of cases), potentially due to its greater length and therefore greater propensity to twist along its long axis compromising its vascular supply [4]. The aetiology of primary omental infarction is unknown. However, proposed contributing factors include redundant omentum, obesity, vascular congestion (right-sided heart failure), increased intra-abdominal pressure, vigorous exercise, and hyperperistalsis due to overeating [2]. Interestingly, the majority of cases present as acute lower right-sided abdominal pain and are often misdiagnosed as acute appendicitis. Here we describe a case of omental infarction mimicking acute cholecystitis. We describe the diagnostic approach and recommend the use of laparoscopy to establish causality when clinical findings and primary patient investigations are inconclusive.

## 2. Case Presentation

A 40-year-old man presented with 7 days of worsening right upper abdominal, epigastric, and right shoulder tip pain aggravated by movement. Past medical history included a similar presentation 6 months earlier, which was investigated with upper abdominal ultrasound. This was unremarkable apart from gallbladder sludge. Physical examination revealed a blood pressure of 159/91 mmHg and heart and respiration rates of 64 beats/min and 20 breaths/min, respectively. He was afebrile with no symptoms of nausea or vomiting, exhibited no change in bowel or urinary output, was haemodynamically stable, and was not medicated for any other conditions. His abdomen was soft with focal peritonism over the medial right upper quadrant and epigastrium, indicating a positive Murphy's sign. Erect chest X-ray and urinalysis were normal. Blood and biochemical analyses revealed mild leucocytosis (WCC 12,100/mm^3^), mildly elevated C-reactive protein (8.9 mg/L), bilirubin (28 *μ*mol/L), and lipase (115 U/L), but was otherwise unremarkable. The patient was commenced on IV fluids and fentanyl (20–50 *μ*g) as required for pain. Triple antibiotics (gentamycin, amoxicillin, and metronidazole) were initiated for presumed cholecystitis and a repeat ultrasound of the upper abdomen was obtained. Upon ultrasonography the patient experienced probe tenderness. Mobile sludge was again identified within the gallbladder, which comprised a normal wall thickness. Although no pericholecystic fluid was observed, a presumptive diagnosis of acute cholecystitis was made.

**Figure 1:**
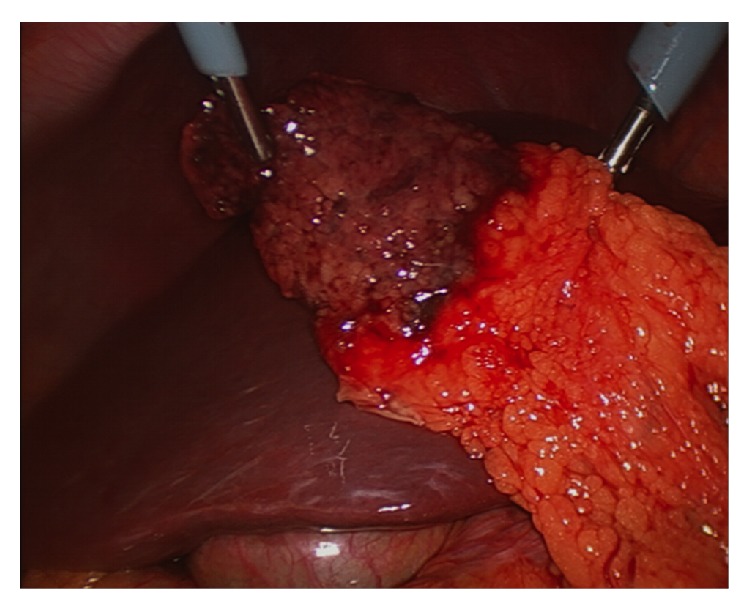
Intraoperative image of the infarcted omental segment following mobilization from the anterior abdominal wall.

**Figure 2:**
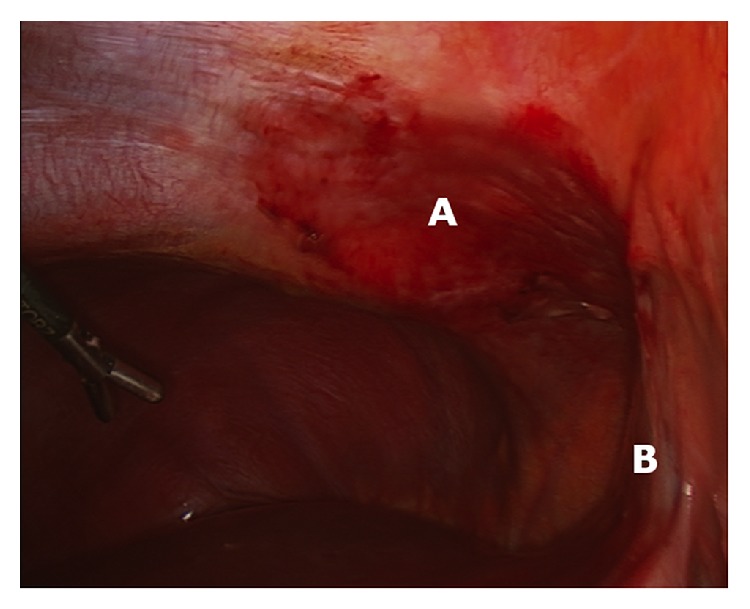
Intraoperative image showing inflammatory changes to the anterior abdominal wall (A) located adjacent to the falciform ligament (B).

The patient proceeded to exploratory laparoscopy, at which the gallbladder was normal in appearance. However, the right-sided greater omentum was partially upturned with a segment wedged between the liver and the anterior abdominal wall, just to the right of the falciform ligament. This segment was grossly necrotic and had adhered to the abdominal wall (Figures 1 and 2). The segment required mobilization and was resected using a surgical stapler. The gallbladder was left* in situ*. Postoperatively the patient was discharged home on day 1, with near complete resolution of his presenting symptoms.

## 3. Discussion

Omental infarction is known to mimic other more common causes of abdominal pain, such as appendicitis or cholecystitis, and approximately 5% of cases are diagnosed nonoperatively [5]. In cases diagnosed radiologically with CT or ultrasound, conservative management is successful in up to 78% of cases, possibly at the expense of a longer hospital stay and higher use of opioid analgesia, relative to early laparoscopic resection [2, 6]. One could consider the use of computed tomography (CT) in cases like this, where there is diagnostic uncertainty and radiation exposure or IV contrast is not a significant issue. At CT scan the most common finding is an ill-defined heterogeneous fat density with surrounding inflammatory changes [7]. Ultrasound is specific but not sensitive for diagnosing omental infarction, with abnormalities detected in less than 50% of cases, even when reviewed retrospectively [8]. However, sonography has a greater yield when differentiating malignant omental deposits [9] and is suitable for guidance of percutaneous biopsies of omental and peritoneal nodules [10]. Our case was not diagnosed before laparoscopy. Although no significant differences in patient outcomes following surgery versus conservative management have been documented, we suggest that primary care physicians consider surgical referral and intervention if intractable symptoms or diagnostic uncertainty exists.

In summary, omental infarction should be considered as an alternate diagnosis for an acute upper abdomen, especially if primary investigations do not fit acute cholecystitis.
